# Self-organization of organoids from endoderm-derived cells

**DOI:** 10.1007/s00109-020-02010-w

**Published:** 2020-11-22

**Authors:** Allison Lewis, Rashmiparvathi Keshara, Yung Hae Kim, Anne Grapin-Botton

**Affiliations:** 1 Max Planck Institute of Molecular Cell Biology and Genetics, Dresden, Germany; 2 The Novo Nordisk Foundation Center for Stem Cell Biology (DanStem), Faculty of Health Sciences, University of Copenhagen, Copenhagen, Denmark

**Keywords:** Intestine, Pancreas, Lungs, Prostate, Liver, Emergence

## Abstract

Organoids constitute biological systems which are used to model organ development, homeostasis, regeneration, and disease in vitro and hold promise for use in therapy. Reflecting in vivo development, organoids form from tissue cells or pluripotent stem cells. Cues provided from the media and individual cells promote self-organization of these uniform starting cells into a structure, with emergent differentiated cells, morphology, and often functionality that resemble the tissue of origin. Therefore, organoids provide a complement to two-dimensional in vitro culture and in vivo animal models of development, providing the experimental control and flexibility of in vitro methods with the three-dimensional context of in vivo models, with fewer ethical restraints than human or animal work. However, using organoids, we are only just beginning to understand on the cellular level how the external conditions and signaling between individual cells promote the emergence of cells and structures. In this review, we focus specifically on organoids derived from endodermal tissues: the starting conditions of the cells, signaling mechanisms, and external media that allow the emergence of higher order self-organization.

**Supplementary Information:**

The online version contains supplementary material available at 10.1007/s00109-020-02010-w.

## Introduction

Organoids are three-dimensional (3D) in vitro systems, which model organs in terms of differentiated cell types and their spatial arrangement, morphology, and functionality, even without auxiliary systems, such as blood vessels, a nervous system, and stromal cells. Thus, organoids are useful tools to investigate organ development, adult tissue homeostasis, and regeneration, as well as disease manifestation and therapeutic avenues. Broadly, we include spheroids, which often harbor a single cell type, as they are a simple and useful system to study progenitor maintenance and differentiation potential, which serves as the foundation of organ and organoid generation.

**Fig. 1:**
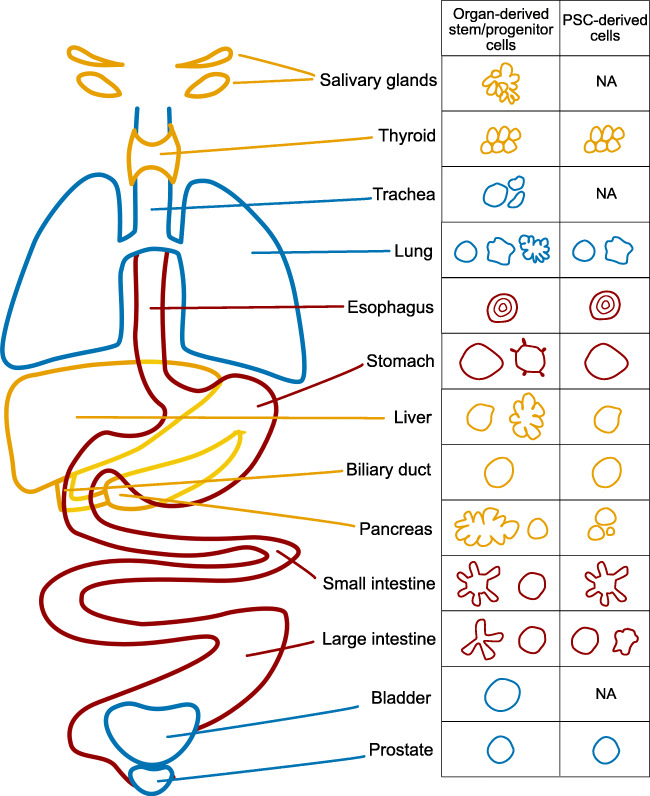
Illustration of endoderm-derived organoids specifying the source of cells, either organ-derived primary cells or PSC-derived cells, that form organoids as well as their representative morphologies. Note that the organoid architecture is not always the same depending of which cell type is used as a source. NA, not applicable means that no organoid of this kind is known to the authors. Representative citations are listed in Table S1

Over the past decade, organoid systems have earnestly developed using both embryonic stem cells (ESCs) [1] and adult stem cells [2]. For example, controlled neuronal differentiation of ESC aggregates generated polarized cortical neuroepithelia, which mimicked different regions of cortical tissue, as well as different zones of neural layers in the cortex [1]. Additionally, adult intestinal stem cells in small clusters or single cells undergo self-renewal, form crypts, and generate villi composed of all differentiated cell types, when exposed to the right culture conditions [2]. These pioneering studies have paved the way for many others which combine self-organization and control by media components—often chosen based on the knowledge of signaling pathways used in development and homeostasis. In this review, we will focus on endoderm-derived organoids such as lungs and trachea [3–10], liver [11–17], bile duct [18–21], pancreas [22–26], intestine [2, 27–32], esophagus [33–35], stomach [36–40], prostate [41–47], salivary gland [48–54], bladder [55], and thyroid [56–58] (Fig. 1; Table S1 providing more specific information regarding source of cells, species, methods, media, etc.). We focus on the non-diseased tissue and do not include gastruloids, though they may contain endoderm, because it is unclear whether it is definitive or visceral endoderm. For reviews on these topics, we refer the reader to articles in this issue by Kai Kretzschmar and Ali Hemmati-Brivalou and colleagues, respectively.

Self-organization is a process by which local interactions between parts of an initially disordered system lead to the formation of higher order structures. This concept, that has found applications in physics, animal behavior, biology, and social sciences, is frequently ascribed to organoids and can be reformulated: self-organization is a process by which local interactions between cells that are initially disordered lead to the emergence of patterns and functions at the scale above, that is the whole organoid. This organization is not driven by a single cell organizing the group or an external control but is expected to have a distributed command over all the components/cells of the system. This distributed command makes the system robust to perturbations allowing for maintenance of homeostasis and self-repair. From studies in other fields, one expects that the process will be spontaneous when sufficient energy is available (in contrast to self-assembly which is a process that does not require energy input), and it will depend on non-linear dynamics rather than linear relations among components/cells as well as feedback control [59, 60]. Positive feedback can lead to the growth of the system and usually ceases when the system reaches a new conformation, with a stable, negative feedback state. These changes of states are highly responsive to the environment of self-organization. Boundary conditions can be imposed on self-organization, which has been done mostly by controlling media components, as well as by the intrinsic properties of the starting cells. Another level of control can be added using environments with different material properties as alternatives to Matrigel [22, 61, 62], or by imposing spatial constraints (see the article by M. Lutolf in this issue). For example, developing two types of organoids such as foregut and midgut organoids and placing them side by side can lead to the formation of a new self-organized structure at the interface, thereby forming the hepato-biliary-pancreatic region [63].

Focusing on endoderm-derived organoid systems, we discuss questions that are relevant to the whole organoid field: what types of cells have the capacity to self-organize? What exchange of signals between cells and scale of interactions are necessary to initiate self-organization and further develop the process? What kind of external controls facilitate the self-organization of organoids?

## The initial disordered cells

An important consideration in self-organization is a good description of the initial parts of the system, the cells. The initial cells used to initiate organoids are very diverse. They can be stem cells or progenitors from an adult or a fetal organ, or pluripotent stem cells that can be engineered to acquire the identity of specific endodermal organs. In some instances, primary cells with the ability to proliferate, either innate or acquired in vitro, have been used to generate organoids. A fascinating feature is that dissociated cells, either alone or as a re-assembled group, can interact to form a tissue- or organ-like structure. This goes well beyond the capacity of an explant to be maintained in culture. Which cells can do this? What are the conditions?

### Organoids from adult stem cells, progenitors, or any cell type with proliferation potential

A common source of cells for organoids is tissue stem cells or progenitors, which have a natural propensity to generate daughter cells that can differentiate. In this regard, the most extensively studied system is intestinal organoids: either single intestinal adult stem cells expressing the marker LGR5 or whole crypts that can form organoids in vitro [2]. Although LGR5^+^ cells from the stomach can also seed organoids [40], LGR5 is not an exclusive marker for tissue stem cells. Several tissue organoids are generated from stem cells that do not express LGR5, for example, in the lungs [3, 4, 8], likely in the trachea [35], prostate [43–47], pancreatic islets of Langerhans [64], and possibly in the salivary gland [48, 49, 65] and bladder [55]. The observation that Troy+ cells from the stomach can generate organoids, together with in vivo experiments, suggests that they may also act as a reserve stem cell population [39]. In many other tissues, there are no LGR5^+^ cells, but cells expressing other markers have been established as stem cells, as for example, in the lung. For some tissues, stem cell markers have not been identified, and in some cases, organ renewal may not be based on stem cells. The evidence that a cell can seed an organoid cannot alone serve as a proof that it is a stem cell. Indeed, any proliferative cell can generate a cluster of daughters.

Illustrating the point above, it is becoming clear that the expression of LGR5 is dynamic and plastic: single intestinal LGR5^+^ cells seeded in vitro downregulate LGR5 during the first day of culture, correlating with the onset of fast proliferation and re-express LGR5 after 62 h [66]. Furthermore, single LGR5− cells can also generate organoids, although with less efficiency compared to LGR5^+^ cells, and upregulate LGR5 within 62 h. This reveals that cells that lose their stem cell marker can generate organoids. In the adult pancreas, LGR5 is not expressed under homeostatic conditions, but is acquired in ductal cells forming spheres [23]. The acquisition of LGR5 by pancreatic spheres in vitro, as well as upon injury in vivo, may be the hallmark of a regenerative response, although comparisons between the processes, including the full transcriptome, should be performed to assess how extensive the similarity is [23]. While the mechanisms controlling this reprogramming/regaining of LGR5 in the intestine and the pancreas are not known, some light was recently shed in the liver about the early molecular changes occurring when a cell is dissociated from its neighbors and seeded in vitro. Aloia et al. [67] revealed transient transcriptional and epigenetic remodeling of differentiated ductal cells in response to organoid initiation and tissue damage, and notably, the importance of the DNA-demethylation protein Tet1. However, whether this is a de-differentiation to a progenitor state that has counterparts in vivo or a direct trans-differentiation remains unknown. Interestingly, it has been proposed that adult intestinal cells seeded in vitro are reprogrammed to a “primitive state” by expressing the markers of the fetal intestine [68]. The gain of proliferative potential, and in some cases, cellular plasticity, may explain why cells of the liver [11, 12, 16, 18] or pancreas [23, 25, 69] can start active proliferation and generate organoids/spheroids, despite proliferating slowly in vivo and lacking stem cell properties (though debated for both tissues). In contrast to intestinal and liver cells, adult pancreatic ductal cells in conditions of organoid formation gain LGR5 and a higher proliferative capacity, but yield only ductal cells in pancreatic spheres [22], which implies that cells with the LGR5 expression may be unipotent.

In addition to adult stem cells, progenitors from the embryonic and fetal tissue also naturally proliferate and give rise to multiple cell types. These progenitors can similarly generate organoids, as shown in the pancreas [64], lungs [6], intestine [70], and liver [71]. The *Lgr5* expression in the developing murine liver signifies a population of bipotent hepatoblasts that can give rise to both hepatocytes and cholangiocytes and can form embryonic liver organoids in vitro [71], whereas in the fetal intestine cells expressing Lgr5 form organoids similarly to those that do not [72], these organoids are different from those generated from adult intestinal cells as they develop spheres without crypts and can proliferate in the absence of Wnt3A [70].

Many organoids can be initiated from a single cell (Table S1) when starting from primary cells, but the probability of organoid formation increases when two or more cells are seeded together [7, 22, 29]. How these cells cooperate to promote organoid formation will be discussed below. Furthermore, single cells are usually inefficient at forming organoids when freshly isolated from the tissue and become more potent after passaging, and for some organs, the presence of other cell types is needed to initiate organoids. Notably, pancreatic islet organoids were produced by co-culturing sorted Procr+ cells with endothelial cells in 3D [64]. Other cell types can also be added even if they are not strictly necessary, such as mesenchymal or endothelial cells [14, 73]. When the system starts from only one cell, more components in the system soon emerge as this cell and its daughters divide. This is different from many self-organized systems such as bird flocking where the number of components in the system is stable at the time scales observed. Additionally, in some organoid/sphere systems, the cells are propagated in a similar state, while in others, asymmetries appear with daughter cells acquiring different identities and properties. This may depend on the potency of the initial cell, and it would be interesting to know in which conditions asymmetry emerges.

### Organoids from pluripotent stem cells

Organoids can also be produced after engineering pluripotent stem cells into endodermal tissues. This followed shortly after those derived from tissue stem cells from endodermal organs [30], and the field has remained essentially focused on producing human organoids. Interestingly, these endodermal organoids are generally produced by a method that is very different from those used in other germ layers, such as kidney or brain organoids, starting with differentiation in 2D rather than initiating differentiation on aggregates of hESCs.

**Fig. 2:**
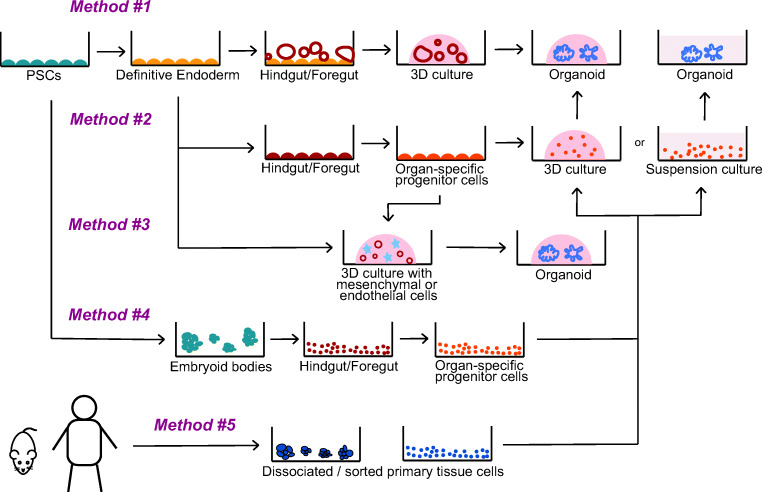
General overview of methods used for generating endoderm-derived tissue organoids. Methods 1–4 depict the generation of organoids from pluripotent stem cells (PSCs). Method 5 schematizes the prevalent method used for producing organoids from primary tissue-derived cells. Representative citations are listed in Table S1

Spence et al. [30] pioneered this culture system, where human pluripotent stem cells (hPSCs) are seeded as a monolayer, and signaling molecules in the culture medium are used to direct their differentiation (see below the section on control by the medium) (method 1 in Fig. 2). Eventually, groups of cells bud out to form 3D hindgut spheres, in a process that is not yet understood. When embedded in Matrigel, the spheres grow to form human intestinal organoids encompassing both intestinal epithelium and its mesenchymal niche, with villus-like domains composed of intestinal stem cells and all appropriate differentiated cell types. Unlike the organoids derived from intestinal crypts, these hPSC-derived organoid cultures contain mesoderm-derived mesenchymal cells which are produced in small numbers during endoderm induction by WNTs and activin. Organoids with mesenchymal cells are potential model systems to understand epithelial-mesenchymal interactions during organogenesis and cell fate decision, as well as tissue niche determination; yet, the presence of mesenchyme appears to be dispensable to form organoids. Mithal et al. [27] were indeed able to generate mesenchyme-free colonic/proximal intestinal organoids from human-induced PSCs (hiPSCs) by sorting a pure population of intestinal progenitors with the NKX2–1^−^CD47^low^ expression and seeding them in 3D Matrigel. These progenitors differentiated to form organoids with proximal/duodenum intestinal markers without any mesenchymal cells. Similarly, sorted populations of hPSC-derived progenitor cells can generate lung epithelial spheroids, alveolar spheroids, and thyroid follicle organoids without any mesenchymal cells [57, 74, 75].

Developmental pathways can be exploited during the differentiation of endodermal cells in 2D [30, 76] by introducing extracellular signaling cytokines in the medium at early differentiation stages. This drives the differentiation into endodermal organs formed at different anteroposterior positions of the body axis—a concept that was further exploited to produce colonic organoids [77], stomach antrum-like organoids [38], and lung organoids [10, 78]*.*

For other organoids, hPSCs can be differentiated into tissue-specific progenitor cells in 2D, and then isolated cells or small groups can be embedded in Matrigel (method 2 in Fig. 2). This method was used to produce pancreatic progenitors in 2D, with subsequent seeding and expansion in 3D in Matrigel [26, 79, 80] or in suspension culture [26]. The effect of suspension versus Matrigel remains unclear, although in epithelial organoids, Matrigel contributes to defining the polarity [26, 81]. A similar method consisting in producing the cells in 2D and subsequently seeding them in 3D in Matrigel was used to produce esophageal organoids [33, 34] and cholangiocyte organoids from hepatoblasts [82].

Another method to produce endoderm-derived organoids consists of deriving definitive endoderm or tissue specific progenitor cells from hPSCs in 2D and co-culturing them with tissue-derived mesenchymal or epithelial cells (method 3 in Fig. 2). This was exploited to produce prostate organoids by co-culture with rat urogenital sinus mesenchymal cells [41]. In a similar way, cholangiocytes with mature ductal phenotype were produced from hPSC-derived hepatoblasts by co-culturing with OP9 stromal cells [19]. In addition, liver organoids were generated by co-culturing hiPSC-derived hepatic endodermal cells with human umbilical vein endothelial cells and human mesenchymal stem cells [14]. This study created a new perspective for hPSC-derived organ transplantation studies, as they could generate functional liver buds with human vasculature upon transplantation into mice.

For some organoids, hPSCs are seeded as 3D aggregates to form embryoid bodies (EBs). The emergence of a well-defined stratified differentiated epithelium from disordered EBs has been demonstrated in esophageal organoids [34]. Thyroid organoids were generated by culturing hPSC-derived endodermal cells as clumps and inducing anterior differentiation (method 4 in Fig. 2). Thyroid progenitor cells were then seeded and differentiated to form mature thyroid organoids [57]. A similar method of seeding endodermal cells as clumps has been employed to produce lung organoids that recapitulate the development of fetal lungs [74, 75].

As illustrated above, definitive endoderm derived from hPSCs can undergo directed differentiation into many tissues, though the methodological principles have so far differed between organs following the choices of different schools of thought.

## What type of order emerges?

### Emergence of spatial order

Systems are said to be emergent when the system components have specific properties not present in the individual parts. In that sense, organoids have emergent spatial organizations (Fig. 1; Table S1) in the same spirit as fish shoals or bird flocks. A very common organization is the formation of a lumen in almost all endodermal organoids reported, but a notable deviation is the tracheospheres, which form a sphere of concentric squamous layers with the most differentiated cells occupying the center [33–35]. In most organoids, a central spherical lumen is formed, while in intestinal organoids, this lumen has positive and negative curvatures following the crypts that form in the subset of organoids that undergo differentiation. In this example, it is important to note that organoids formed only from enterocytes have a spherical lumen, which indicates a correlation between differentiation and shape [66]. Though a vast majority of organoids form a spherical lumen, the structure of the epithelium lining the lumen can be different: either a monolayer of squamous or cuboidal cells (lung alveoli [7]), simple columnar (intestinal [30]), pseudostratified (lung airway spheres [3, 8]), bilayered (prostate [41–47]), or stratified (cuboidal, columnar, or squamous). An extreme and unusual type of lumen is found in the pancreas. In pancreas organoids derived from the embryonic tissue, it was observed that the same cells embedded in Matrigel but cultured in different media formed either spheres lined by a monolayer of cells or more complex organoids harboring an inner network of ducts [22, 83]. Thyroid organoids represent another interesting type of morphogenesis where a grape of unconnected follicles appear to form [57]. Importantly, these different types of lumen are in vitro phenomena relevant to the diversity of lumen (and epithelia lining them) seen in the different organs. Why organoids form such different types of lumens remains to be investigated. This lumen formation is a self-organizing behavior, as cells collectively polarize but do not do so as individuals.

Another type of structure seen in organoids is the presence of branches or folds. While most organoids reported in endoderm are roughly spherical, differentiated intestinal organoids exhibit local folds forming crypts, which coincide with Paneth and Lgr5^+^ stem cells [2]. Pancreas organoids derived from embryos similarily exhibit folds but they are more numerous, and their structure is different, reminiscent of the branches seen in vivo. In this case too, there is a correlation with differentiated cells, as acinar cells are seen in the outer folded areas [22]. In these two systems, differentiated cells can be seen before morphological changes (Paneth for intestinal organoids and acinar cells for pancreas organoids), and it is postulated that the folds are a consequence of differentiation. Folds have also been seen in lung organoids derived from embryonic tip cells, which eventually form bubble-like structures (alveoli) at the tips [6, 7]. How the folds form remains to be investigated. In the case of the pancreas, the emergence of folds was seen after roughly 5 days of culture and correlated better with the size of organoids rather than time in culture [84]. Experiments on brain folds suggest that an interplay between the material properties of the organoid and the environment where they are grown is an important determinant of fold formation [85].

An important difference of organoids from organs found in vivo is the limited complexity of shapes and their usually isotropic nature. Though there are a few examples of tube formation [41], many intestinal organs normally forming tubes form spheres instead. Although the pancreas is an elongated organ, progenitors form roughly isotropic pancreatic organoids [22]. However, there are a few exceptions such as gastruloids, which elongate in a self-organized manner [86]. In this example, the mechanisms breaking symmetry are likely driven by the formation of a node, but why they lead to an elongation (as seen in the mammalian embryo) rather than an isotropic movement (reminiscent of frog gastrulation) is unknown. Anisotropies can be triggered by engineering assemblies of organoids which lead to the formation of more complex anisotropic shapes. For example, the fusion between foregut and hindgut organoids leads to the formation of a midgut and a complex structure reminiscent of the hepato-pancreato-biliary region at the interface [63]. The last category which will likely expand in future years is the directed control of shape by environmental constraints (see the article by M. Lutolf in this issue).

### Emergence of differentiation

In addition to morphogenesis, new cell types often emerge in organoids (Table S1). The emergence of diversity from initially identical parts has also been studied in other emergent systems, such as colonies of social insects [87–89]. Such models typically utilize positive feedback signals given to agents of the same caste in order to encourage the emergence of specialization for a specific task [89] and negative feedback to balance the different types of agents. These signals can operate with thresholds [88]. We know little about such feedback systems in the organoid field. The differentiation of organoids depends largely on the potential of the cells seeded, the ability of the medium to reprogram them to a stem/progenitor state (if they are not multipotent), and the presence of components promoting and sustaining the differentiation state. Defining these media has been a major focus in the organoid field. Very often media have been designed to either maintain the progenitor state or promote differentiation, and these media can be applied sequentially to initially promote organoid growth and then control composition. When starting from tissue stem/progenitor cells from a given organ, the cell types that are expected to differentiate do so according to the tissue of origin, although the ratios are not always faithful to the original organ. It has notably been difficult to obtain endocrine cells in the intestine [90], lungs, and prostate [41, 46, 47], or parietal and enterochromaffin cells of the stomach [37]. An overview of differentiation in different organoid systems is provided in Table S1. For some organs, though individual cell types can be generated, combining them in a single organoid remains a challenge, as for example, cholangiocytes and hepatocytes in the liver (Table S1).

Organoids derived from hPSCs pose an additional problem, which is the generation of “unwanted” cell types, though this is under investigated. Based on the engineering of desired cells driven by their exposure to a sequence of media components, cells rarely commit with 100% efficiency, and the presence of other cell types should be thoroughly investigated. The use of single-cell sequencing, which can detect rare populations, will be suitable for this purpose.

### Emergence of functional properties

Organoids are often generated with the hope that they can serve as models of organ function and its impairment by diseases; therefore, the occurrence of emergent functional properties (Table S1) is an important feature to consider. So far, only a handful of functions have been explored. For example, the pumping function was assessed in intestinal, lung, pancreatic, and cholangiocyte organoids and its impairment in cystic fibrosis [8, 19, 26, 27, 91]. Other examples include glucose stimulated insulin secretion [64], response to androgens for prostate [43, 47], acid secretion for stomach organoids [36], amylase secretion for pancreas, and salivary organoids [26, 49, 65], as well as saliva secretion for the latter [49, 65]. Some level of functionality was demonstrated for liver organoids, notably the ability of hepatocytes to take up low-density lipoproteins, store glycogen, secrete albumin into the lumen, and the presence of detoxifying activity—though to a lesser extent than freshly isolated hepatocytes [12, 16]. Many more functions remain to be explored, and an important comparison with endogenous organ functions has to be performed to assess the limitations of the models [82].

Particularly for organoids derived from hPSCs, the cells are engineered to differentiate according to a process that takes 9 months in the body. This is recapitulated in vitro in a few weeks, though culture over months is becoming possible. Whether the process is sped up in vitro or if the cells follow their natural timing and remain early fetal cells is a question that is starting to be addressed. When investigated, it has been argued that they remain fetal [13, 14, 30, 33, 34, 38, 74, 75, 77, 78, 92], but the developmental timing in vitro remains somewhat of an enigma. Only a few studies have addressed the maturity of cells systematically by comparing those generated in vitro to their fetal and adult counterparts [13]. This is also relevant for organoids derived from adult organs which may reset their clock to developmental stages, though this needs to be addressed with full genome comparisons and appropriate benchmarks [68, 93, 94].

### Emergence of organ domains

In the organoids that acquire a complex organization, it is observed that organ domains can emerge at a defined spatial location in patterns reminiscent of those observed in vivo (Table S1). For example, pancreas organoids derived from the fetal tissue segregate acinar cells at the periphery [22], crypts harboring stem cells form in intestinal organoids [66], and pits with pit cells develop in stomach organoids [37, 40]. These processes can combine local signaling activities but may also result from cell rearrangements and tissue mechanics that will be clarified when live imaging becomes more widely used [66].

#### Exchange of signals between cells and scale of interactions

An important principle in self-organization is the exchange of information between the components of the system. We have highlighted in the previous paragraphs that organoids start from either a single cell or multiple cells. In the case of singlets, the exchange of signals between cells can in principle start as soon as the first cell has generated two daughters. In this scenario, the two cells may be the same, or asymmetric cell division could be the first event leading to symmetry breaking, though it was so far seldom documented and will need further investigations [95].

**Fig. 3:**
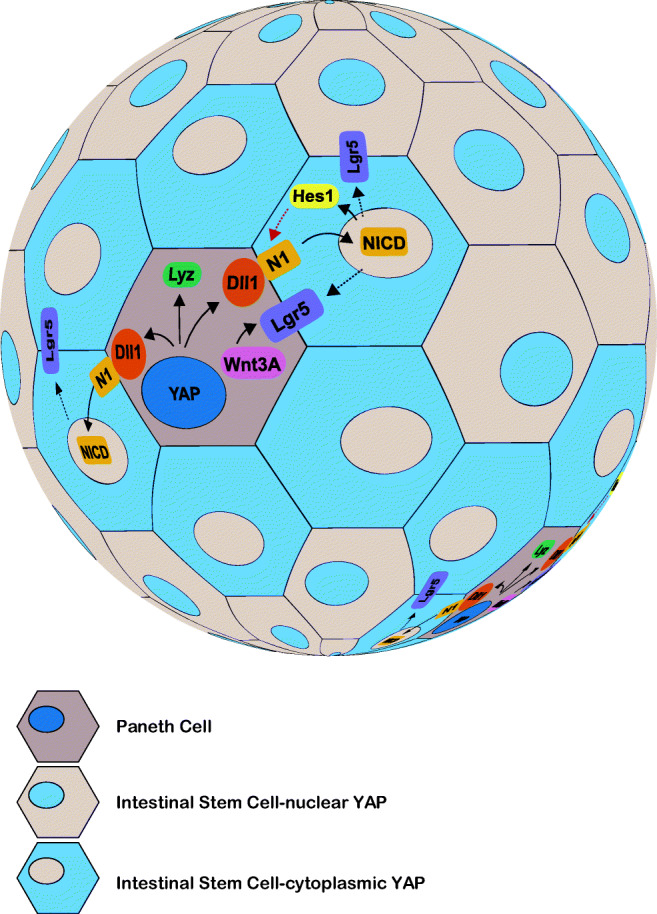
YAP-Notch signaling cross talk promotes Paneth cell differentiation and intestinal stem cell niche reestablishment. At the onset of the culture, intestinal stem cells lose the Lgr5 expression and have uniform YAP (blue) nuclear localization. Variability in YAP cellular localization occurs with organoid growth and when a critical cell number is reached, the first Paneth cell emerges and expresses lysozyme (Lyz) and the Notch ligand delta-like 1 (Dll1) which activates the Notch1 (N1) receptor on neighboring cells. These cells lose YAP nuclear localization and re-express Lgr5, making them competent to receive Wnt signals from Paneth cells. Lgr5 re-expressing cells express Hes1: a readout for active Notch signaling, which may contribute to Notch lateral inhibition with the neighboring Paneth cell forming a negative feedback loop to maintain its identity

Recent experiments in intestinal organoids have shed light on the signals that cells exchange to self-organize after tissue dissociation. Though single Lgr5^+^ stem cells from the adult mouse intestine can generate organoids [2], *Lgr5*^+^ intestinal stem cell-Paneth cell doublets exhibit a 12-fold increase in organoid formation efficiency, compared to that observed from culture of single *Lgr5*^+^ cells or even multiple *Lgr5*^+^ cells [29]. Furthermore, genetic loss of Paneth cells in vivo results in a corresponding decrease in intestinal stem cells [29]. These data revealed that Paneth cells supply essential niche signals for the self-organization of crypts and villi both in vitro and in vivo. This study further showed that the secretion of Wnt ligands by Paneth cells, including but not necessarily exclusively Wnt3, signaled to LGR5^+^ cells triggering expansion (Fig. 3). Wnt signals from Paneth cells [29] (or from telocytes in vivo) maintain LGR5 in intestinal stem cells, and they enable Rspondin1 to bind LGR5 receptors which promotes proliferation of intestinal stem cells [96]. It is hypothesized that in vivo, as intestinal stem cells proliferate, they titrate out Wnt signals to restrict proliferation as the distance from the Paneth cell increases (Fig. 3).

This dependence on a multicellular starting point is not restricted to intestinal organoids and is seen in organoids derived from embryonic mouse pancreas [22]. It was hypothesized that this may reflect the minimum number of cells required to maintain Notch signaling, which is important for pancreas progenitor maintenance and expansion in vivo. In contrast to the intestinal organoids, the presence of secretory endocrine cells akin to Paneth cells was dispensable to form pancreatic organoids. However, the presence of *Neurog3*-expressing endocrine progenitors correlated with an increased efficiency of organoid formation/expansion. Since both endocrine progenitors and endocrine cells express Notch ligands, the role of Notch signaling was further investigated. Smaller organoids were observed upon Notch inhibition, which is consistent with the known role of the pathway in progenitor expansion. In contrast, increasing Notch signaling through genetic manipulation of the starting cell population did not allow organoid formation from single cells. The role of Notch signaling, and whether endocrine progenitors rather than endocrine cells send signals enabling the proliferation in neighboring progenitors, would deserve further investigations. Moreover, evidence for cooperativity and communication between epithelial cell types in other organoid systems is scarce but would deserve more investigations.

In many systems, single cells can form organoids; yet, this by no means excludes the importance of cooperativity. Indeed, Wnt ligands can be provided in the culture medium as a surrogate for the signals normally provided by other cells, such as Paneth cells and telocytes, to promote the formation of organoids from single intestinal stem cells [29, 97]. The components of the media can thus alleviate the needs for signal exchange between cells during self-organization. It is also becoming evident that in some organs, providing non-epithelial cells can enable organoid growth from single epithelial cells. For example, it was recently found that a rare population of Procr^+^ islet cells could form organoids in the presence of endothelial cells [64], though the signals involved and whether they are diffusible or require contact have not been investigated.

The examples above illustrate the concept that interactions between cells can be important for organoid initiation and can be mimicked by including the signals in the culture medium. However, a recent study shows that we have so far only scratched the surface, and sequential signaling events, likely combined with mechanical cues, operate during the early stages of organoid formation (Fig. 3) [66]. In intestinal organoids, despite being established from *Lgr5*^+^ intestinal stem cells and the presence of its agonist WNT3A in the medium, cells first lose the *Lgr5* gene expression at the onset of the organoid culture. It is not yet clear why this loss happens despite the presence of appropriate stimuli, as the first day of culture has not been further analyzed. However, at 24–36 h of culture, when the organoid has a few cells, YAP cellular localization changes from uniformly nuclear to become variable with translocation to the cytosol in some cells at 48 h. This cell-to-cell variability in YAP localization was the first sign of symmetry breaking and found to be essential for Paneth cell differentiation, as either homogenous nuclear or cytoplasmic YAP localization results in an unbranched, spherical organoid composed uniformly of enterocytes. Further analysis revealed that cells with nuclear YAP also subsequently expressed the Paneth cell marker lysozyme and the Notch ligand Dll1, which was shown to be a YAP transcriptional target. It is hypothesized that the expression of Dll1 triggers Notch signaling in neighboring cells (indicated by the expression of the transcription factor HES1) which is known to be important for intestinal stem cell proliferation [98, 66, 99] (Fig. 3). Notch signaling also promotes the transcription of the stem cell marker *Lgr5,* although it is unclear whether it is direct or even cell autonomous, as Notch signaling inhibition results in loss of the Lgr5 expression, yet Hes1 knock-out mice do not lose the expression of the stem cell marker [98]*.*

A role for YAP in promoting the reversion of adult tissues to a fetal identity is also emerging in several tissues and organoids. For example, work combining in vivo and organoid-based experiments have demonstrated that in vivo ectopic YAP activation results in the de-differentiation of hepatocytes to a fetal progenitor identity via Notch signaling. These de-differentiated cells could then be used to generate organoids. This function of YAP seems to hold true in other tissues, including mammary gland, neurons, and pancreatic acini. In these systems, the ectopic expression of YAP allows differentiated cells to revert to a progenitor-like identity with proliferative capacity to form organoids composed of differentiated cell types [100]. This potential scenario could also be at play in intestinal organoids, where the intestinal stem cells are defined by activated YAP signaling [66, 99]. Moreover, in the colon, seeding epithelial cells in vitro appear to mimic a regenerative response characterized by activated YAP and a fetal identity [68]. In the developing mouse esophagus in vivo and in organoids, progenitor cells maintain YAP nuclear localization, whereas in differentiating suprabasal cells YAP has relocalized to the cytoplasm. Both inhibition of YAP and its activation reduce stratification, suggesting that cell-cell variability is also important in this example [101]. However, experiments in the liver show that YAP does not systematically promote a fetal progenitor identity, as its activation in bile duct cells results in cell proliferation with an absence of the progenitor activation [102].

What triggers the initial nuclear translocation of YAP when intestinal cells are seeded in vitro—and its reactivation in a subset of cells—remains to be determined [66]. The YAP/TAZ pathway is known to be mechanosensitive, and it is suggested that cell crowding may underlie the observed changes in YAP cellular localization as organoid culture progresses. Moreover, a recent investigation showed that integrin signaling, mediated by changes in the ECM, results in induction of YAP/TAZ target genes, and impairment of this signaling axis results in reduced organoid formation efficiency [68]. These experiments suggest that organoid formation follows a regenerative response, whereby ECM destruction from tissue dissociation activates YAP signaling, which then becomes restricted in some cells and provides the variability necessary for cell differentiation. The YAP activity may become restricted through the restoration of the ECM, and thus regions that achieve this repair more quickly are the first to lose the YAP activity due to the loss of activation by the ECM-integrin signaling axis. It is also possible that the downregulation of YAP occurs after Paneth cell differentiation, perhaps driven by the expression of the ECM protease MMP7. Recent experiments have revealed that inclusion of the inflammatory cytokine TNF-alpha during culture (probably mimicking pro-regenerative signals) increases organoid formation efficiency with a corresponding localization of YAP to the nucleus, suggesting that YAP may also receive inputs from medium components [16]. These experiments also open the door to further investigations on the importance of mechanical processes during organoid formation and their control by the surrounding matrix and neighboring cells [103, 104].

#### Positive and negative feedback loops

There is a long way to go from the few signals we know are exchanged between cells to an understanding of self-organization and emergence in organoids. Though organoids have in promising systems to study self-organization, they lag behind other systems where emergence has been studied. In the paragraphs below, we discuss what has been learned in other self-organizing systems and draw parallels to what may be expected in organoids.

Merely having interactions between cells is not alone sufficient to guarantee emergent behavior; many of the interactions may be negligible or irrelevant. They may cancel each other or even hinder the emergence of interesting behavior by creating “noise.” Thus, it is not just the sheer number of connections between components which encourage emergence, but how these connections are organized. A hierarchical organization is one example that can generate emergent behavior. A common feature of emergent systems is the presence of both positive and negative feedback loops. In general, negative feedback stabilizes structures, whereas positive feedback promotes change [59, 60]. Reinforcement may depend on the absolute number of neighbors (in organoids, neighboring cells) exhibiting a behavior, or perhaps on a specific proportion of the components with the feature (proportion of cells in a given state). Synchronization of responses can also be important for self-organization in animal behavior [105]. In some cases, the system must reach a combined threshold of diversity, organization, and connectivity before emergent behavior appears. The emergent behavior may also need to be temporarily isolated from other interactions before it reaches enough critical mass to self-support. In organoids, domains of emergence may thus exist. An example of temporal control is dual-phase evolution where interactions are applied intermittently, leading to two phases: one in which patterns form or grow and the other in which they are refined or removed [106]. The emergent behavior may happen best in systems poised near criticality where information is propagated through the system rapidly via local interactions with virtually no loss [106]. Small and quick changes in the connectivity between elements can promote the emergence of criticality (percolation theory of information through the network) [106]. In the case of organoids, the coupling between cells may change with time with cell movements and/or the onset of expression of signals, receptors, or their modulators. It is thought that in animal behavior, speed in an adaptation is increased by each element responding not to a change but to its projected future state (projected future position and velocity) [105]. Linking changes in the system to its current state with other states at other time points may thus become important in organoids too.

We also know from other systems that the signal initiating emergent behavior can be external or noise. For example, the Rayleigh-Benard convection emerges only at a specific difference in temperature between the upper and lower layers [59, 60]. These are the so-called boundary conditions. As another example in the living world, when a falcon attacks a flock of starlings, the flock reorganizes [107]. Studies in fish show that some parts (individual fish) are responders to the external perturbation and the others “copy” the behavior. Each part responds to the proportion of other parts linked in the network by defined cues (space occupied on retina, log of distance…) [107]. In many cases, the system is also designed to integrate multiple external stimuli. In schooling fish, for example, the network of interactions is designed to reduce correlated (non-independent) information. These theoretical considerations are important when considering external control, such as the components included in the medium and the temporal exposure to those components, to direct the organoid response and make systems more reproducible.

#### External control

As discussed earlier, cells in organoids not only respond to but also generate and propagate multiple layers of signals, both autonomous and non-autonomous. During the self-organization process, cells selectively adapt to the mixed, available signals provided in culture medium (Table S1). These extrinsic signals contribute to the trajectory for morphogenesis and also initiate the differentiation of progenitor cells. The majority of culture media for endodermal organoids are composed of basal media providing basic nutrients for cell maintenance in culture, such as DMEM or IMDM, including sources of energy. In the early days, FBS was used, but it has been replaced by defined supplements such as B27 or N2. In addition to these components, growth factors and signaling molecules have been essential components for proliferation and maintenance of progenitors. FGFs, EGF, and WNT agonists are the most common growth factors used in organoids; FGFs are seemingly most essential, especially to maintain proliferating progenitors. In most organoid systems, different media are used to sequentially promote proliferation and subsequent differentiation. Common components enabling differentiation are Notch antagonists, TGFβ inhibitors, and BMPs, but organ-specific differentiation signals are also used (Table S1). Organoid culture media serve as a buffet signaling molecules, and yet, cells in organoids help themselves to different dishes as needed during the self-organization process. It would be intriguing to investigate how these homogeneous media components are selectively utilized by multiple cells to generate heterogeneous cell types as well as variable morphology. The extent to which the medium components similarly reach the center of organoids as they grow would deserve more investigations as gradients could be a source of heterogeneity between the external and central cells.

Extracellular matrix (ECM) serves as a scaffold for the cells to develop into organoids which mimic in vivo organ development and structure. In an early salivary study, spheres were cultured in suspension [52, 53], but the subsequent studies adopted ECM-based gels such as Matrigel [52]. Most of the organoid systems to date are cultured in 3D Matrigel or similar scaffolds, which has proven most robust compared to other matrices, though collagen gels have also been successfully used in some instances [108]. Matrigel combined with the fine-tuned selection of growth factors has successfully replaced indispensable mesenchymal cells in many organoid systems, such as lungs, intestine, and salivary organoids. However, there have been numerous efforts to replace Matrigel with better-defined matrices, such as polymers, hydrogel, and/or ECM-functionalized materials [22, 61, 62, 109–113] (see more information in the article by Lutolf in this issue). These matrices provide both a physical niche with defined material properties for the organoid cells to sustain their architecture, and a signaling niche serving as a reservoir for growth factors and signaling molecules provided in the culture media and those secreted by the cells to initiate self-organization. The cells also produce and assemble their own ECM to further facilitate their self-organization.

In addition to the control by the medium and by the matrix, recent developments of organs-on-a-chip systems provide platforms enabling miniaturization of culture, sequential exposure to different medium components, and the automation of readouts of organoids as physiological models [114]. These systems enable the production of organoids with less variability in sizes, built-in readouts for functionality tests, and are useful tools for drug screening and disease modeling. Moreover, body-on-a-chip systems are being established to enable multiple organoids in a single device to investigate interactions and physiology of the whole body [115].

## Conclusion and outlook

The last 10 years have seen the development of organoid systems for most endodermal organs and the refinement of methods for more reproducible systems. Nevertheless, there remain enormous expectations on the field to provide models of development, regeneration, physiology, and diseases. In the coming years, developments towards an architecture closer to endogenous organs and incorporations of multiple cell types—including the vasculature and of automated culture systems—are expected. At the same time, more extensive benchmarking to endogenous organs is necessary to probe the relevance of the models and their limitations. This should enable the field to move from building systems to using them and to making more discoveries rather than re-discovering observations already made in vivo. In addition to providing organs that can be studied, organoids are expected to be useful to explore the mechanisms that enable organs to self-organize. Self-organized systems in the biological and non-biological world will be a great source of inspiration in that respect.

## Supplementary Information

ESM 1 (DOCX 133 kb)

## Data Availability

This article reviews literature and therefore does not contain any associated data and materials.
