# Supplementary material for: Self-organization of organoids from endoderm-derived cells
Source: J Mol Med (Berl). 2020 Nov 22;99(4):449–62. doi: 10.1007/s00109-020-02010-w (PMC8026476; doi:10.1007/s00109-020-02010-w)
Supplement: Supplementary file 1 — (DOCX 133 kb) [file 109_2020_2010_MOESM1_ESM.docx]

**Table S1: Selection of organoid systems for different organs**

| **Tissue** | **Starting cells** | **Method** | **Basal medium** | **Supplement** | **Cytokines/GFs** | **Differentiation and benchmarking to *in vivo*** | **Morpho-genesis** | **Functionality and applications** | **Refe-rences** |
| --- | --- | --- | --- | --- | --- | --- | --- | --- | --- |
| Intestine | Whole intestinal crypts. Single Lgr5+ ISCs from adult mice. | #5 Matrigel | Advanced DMEM/F12 (ethanolamine, glutathione, ascorbic acid, insulin, transferrin, AlbuMAX® II lipid-rich BSA for cell culture, and trace elements sodium selenite, ammonium metavanadate, cupric sulfate, and Mn chloride) |  | EGF, R-spondin 1, Noggin [single cells: Jagged-1 peptide in Matrigel + Y-27632) | Microarray comparison to freshly isolated crypts. ICC showing enteroendocrine cells (CHGA), Paneth cells (LYZ), enterocytes (VIL), and goblet cells (MUC2). | Organoids with crypts and villi | - | [1,2]. Similar medium used by [3,4] |
| Intestine | Dissociated fetal human and postnatal mouse intestine. Sorted Lgr5+ and Lgr5- mouse postnatal intestinal cells. hiPSC-derived posteriorized endoderm aggregates. | #5 Matrigel for tissue. #2 for hiPSCs | Sato 2009 for tissue. Spence 2010 for hiPSC. | Sato 2009 for tissue. Spence 2010 for hiPSC. | Sato 2009 for tissue (=PGE2 for fetal human tissue). Spence 2010 for hiPSC plus PGE2. | ICC and microarray to show absence of differentiated cell types in spheres. qPCR showing Intestinal stem cells (*Lgr5*) and goblet (*Muc2)* and Paneth cells (*Lyz*) in organoids. | Sphere from human fetal intestine, mouse intestine before P2, Lgr5- intestinal cells, and hiPSCs. Organoid with crypts and villi from mouse after P15 and Lgr5+ intestinal cells. | - | [4] |
| Intestine | Single Lgr5+ and Lgr5- ISCs from adult mice. | #5 Matrigel | IntestiCult™ Organoid Growth Medium (Stem Cell Technologies) |  |  | Spheres with enterocytes shown by ICC (Aldolase B) and organoids with enterocytes (Aldolase B), Paneth cells (LYZ) and intestinal stem cells (LGR5-GFP). Bulk RNA-seq for *Lgr5* (ISCs), *Apoc3* (enterocytes), *Lyz1* (Paneth cells), *Muc2* (Goblet cells), *Sst* (enteroendocrine cells in organoids). | Organoids with crypts (villi not mentioned) and spheres with only enterocytes. |  | [3] |
| Intestine | hESCs/hiPSCs --> endodermal floating gut spheres | #1 | DMEM/F12 | Glutamine, EPEPS, N2, B27 | R-Spondin1, Noggin, EGF (for gut spheres: FGF4, WNT3A) | ICC of intestinal markers-proximal and distal intestine (KLF5, CDX2, SOX9). ISH of crypt stem cell markers (LGR5 and ASCL2). ICC of enterocytes (VIL), goblet cells (MUC2), Paneth cells (LYZ), and enteroendocrine cells (CHGA) | Organoids with crypts and villi, mesenchyme | - | [5]. Similar medium used by [4] |
| Intestine | hiPSCs --> CDX2+ intestinal progenitors by dual-smad inhibition (besides lung) | #2 | DMEM/F12 [RPMI] {IMDM/F12} | B27, N2 , HEPES, Glutamax [FBS] {B27, N2} | Noggin, R-Sondin, EGF, CHIR [CHIR, FGF4] {CHIR, KGF, dexamethasone, cAMP, IBMX} | Intestinal marker CDX2 by RNAseq and ICC. ICC showing enteroendocrine cells (CHGA), Paneth cells (LYZ), enterocytes (VIL), and goblet cells (MUC2). qPCR for more markers. | Spheres with sporadically differentiated cells with regional specificity, but no well-defined crypts or villi. | Cystic fibrosis  modeling | [6] |
| Colon | EPCAM+EPHB2+ cells from human colectomies, including clonal. | #5 Expansion or differentiation media, in Matrigel | Advanced DMEM/F-12 | Glutamax, HEPES, B27, Nicotinamide, N-acetyl-Cys, | WNT3A, RSPO1, EGF, Noggin, Gastrin, LY2157299 (TGFbR1 inhibitor), SB202190 (p38 MAPK inhibitor), PEG2 | Stem cells and proliferative progenitors upon expansion, absortive and secretory cells upon differentiation shown by ICC for KRT20 (epithelial), ANPEP, FABP1 and CA1 (absortive), MUC2, TFF3 (secretory). Mucous vacuoles or brush borders by electron microscopy. | Spheres | - | [7] |
| Colon | hPSC --> CDX2+ spheroids | #1 | DMEM/F-12 | N2, B27, HEPES, glutamine | EGF, BMP | Mostly progenitors, but colon-specific enteroendocrine cells upon NEUROG3-induction and BMP-induced mesenchyme. ICC of colonic specific marker SATB2 | Fused spheres | In vivo transplantation for differentiation and morphological resemblance to colon (kidney capsule) | [8] |
|  |  |  |  |  |  |  |  |  |  |
| Stomach | Mouse adult LGR5+ pyloric gland stem cells, clonogenic | #5 Matrigel | Advanced DMEM/F12 | N2, B27, nAcetylcysteine | Gastrin, EGF, R-spondin1, Noggin, FGF10, WNT3A-conditioned media (Exendin 4 for EE differentiation) | ICC showing gastric epithelium (Gif, Muc6, PepC) with stem cells (Lgr5+), pit cells (Muc5ac), neck cells (Tff2) and enteroendocrine cells (ChgA) in differentiation conditions. | Spheres. With time, in differentiation conditions glands form as small outpocketings around the sphere. | - | [9] |
| Stomach | Troy+ or Mist1+ chief cells of murine gastric corpus, clonogenic | #5 Matrigel | Advanced DMEM/F12 | N2, B27, nAcetylcysteine | EGF, Gastrin, FGF10, Noggin, Wnt3a, and R-spondin | ICC for Chief cells (Gif, Troy, Mist1), mucous neck cells (GSII). Pit cells (Muc5ac) in differentiation conditions. Transcriptome. | Spheres | - | [10] |
| Stomach | hESC-derived posterior foregut spheroids | #1 | Advanced DMEM/F12 | N2, B27, Glutamine, HEPES, | EGF, RA, NOG | qPCR and ICC for surface mucous cells, antral gland cells,  gastro endocrine cells, gastric stem cells (SOX2, PDX1, MUC5AC, SOX9, MUC6, LGR5,SYP, GAST, SST, GHRL, 5-HT) | Complex folded and glandular architecture | H. pylori infection | [11] |
|  |  |  |  |  |  |  |  |  |  |
| Lung | hESCs/hiPSCs --> NKX2-1+ progenitor cells | #4 | complete serum-free differentiation media (CSFDM) |  | CHIR, FGF10, KGF, (Y27632 for 1st 24 hours) | Developing lung epithelial markers. qPCR and ISH for lung-specific genes, and ICC for lung markers (SFTPB, MUC1). | Spheres |  | [12] |
| Lung | hESCs/ihiPSCs --> NKX2-1+ progenitors | #4 | cSFDM (IMDM/F12) | B27, N2, BSA, Glutamax, ascorbic acid, MTG | CHIR, KGF, dexamethasone, 8-Br-cAMP, IBMX, (Y27632 for 1st 24 hours) | Fetal cells that mature in culture (though not adult). qPCR for distal lung genes, and ICC of lung markers (EPCAM, NKX2-1, proSFTPB, proSFTPC). Lamellar bodies. | Spheres | Putative alveolar epithelial type 2 cell (distal alveolar cell) producing and secreting surfactant. Alveolar disease  (SFTPB mutation) modeling | [13] |
| Lung | hESCs -> foregut spheroids -> 3D culture | #1 | Advanced DMEM/F12 | N-2, B27, Hepes, Glutamine | Noggin, SB431542 | qPCR for proximal lung genes and ICC showing stratified epithelial cells basal cells (p63), ciliated cell markers (FOXJ1), club cells (SCGB1A1, CC10), alveolar progenitors including AECII (SFTPC, SFTPB, SOX9) and AECI (PDPN, HOPX, SOX9), both proximal and distal airway-like epithelia, mesenchyme (VIM) & myofibroblasts (PDGFRα, Sma). Lamellar bodies. The cells remain fetal in nature: rare SCGB1A1+ cells do not resemble mature club cells, club cells not multiciliated. Benchmarking to fetal lung tissue by RNA-Seq. | Spheres/  organoids |  | [14] |
| Lung | Mouse CD31_CD45_EpCAM+Sca1_ enriched for AT2 cells, or CD31_CD45_EpCAM+  Sca1+ enriched for putative bronchioalveolar stem cells (BaSCs), | #5 + lung endothelial cells | DMEM/F12 | 10% FBS, HEPES, insulin/transferrin/selenium | Several individual growth factors tested | Three colony types arose in BASC cultures: bronchiolar colonies with cells CCSP+, ciliated cells and goblet cells (MUC5AC); alveolar colonies expressing SPC; and mixed morphology structures (bronchioalveolar) colonies containing CCSP-positive and SPC-positive cells. AT2 cells only formed alveolar structures expressing SPC | Organoids with complex shapes, including tubes and sacs |  | [15] |
| Lung bud  tip progenitors | human epithelial tips from 5-19 pcw  lungs as well as 5-9 bronchial stalks. Mouse E12.5 lung bud tips. | #5 | DMEM  PneumaCult for airway differentiation | Hepes, N2, B27 (-VitA), N-acetylcysteine | EGF, FGF7, FGF10,  NOG (Noggin), RSPO1 (R-spondin 1), a GSK3b inhibitor CHIR99021 and a TGFb inhibitor SB431542.Alveolar differentiation medium: (CHIR99021,  FGF7, FGF10, Dexamethosone, cAMP, IBMX, T3, DAPT | Lung tips (SOX2, SOX9, NKX2.1) by ICC as well as transcriptome benchmarked to human fetal material. Transplantation and differentiation in immune-compromised mice injured with a low dose of bleomycin. In airway differentiation conditions: TP63+ basal cells, MUC5AC+ goblet cells and rare ACT+ ciliated cells and mesenchymal cells. In alveolar differentiation, with mesenchymal cells: SFTPC, HTII-280, HOPX and PDPN co-expression and NKX2-1 | Spheres for 6–8 days and then branched; by day 14 |  | [16] |
| Lung bud  tip progenitors | hESCs ->foregut spheroids -> 3D culture as well as human fetal lung bud tips | #1 and #5 | DMEM/F12 | N2, B27, Glutamax, BAS, ascorbic acid, monothioglycerol | FGF7, CHIR, RA | Numerous markers tested at different stages. At the end of differentiation: qPCR and ICC for lung bud tip progenitor markers. club cells, goblet cells (MUC5AC,MUC5B), secrete mucous, bud tip progenitor cells basal cells (p63), ciliated cell markers (FOXJ1), club cells (SCGB1A1), neuroendocrine cells (SNY, CHGA) alveolar progenitors including AECII (SFTPC, SFTPB, ABCA3) and AECI (PDPN, HOPX). Electron microscopy shows lamellar bodies). No P63, FOXJ1, or cilia. Transcriptome comparison to human lung bud tips. | Spheres for human fetal lung-tip; organoids from hPSCs then spheres from their tips. |  | [17] |
|  |  |  |  |  |  |  |  |  |  |
| Trachea and airway epithelium | Mouse primary trachea basal cells and human bronchial basal cells | #5 50% Matrigel at air-liquid interface | DMEM/F12 | HEPES, NaHCO3, Glutamine, bovine pituitary extract, FBS | Insulin, trasferrin, cholera toxin, EGF, RA | ICC for p63 (basal cells), K14 (basal cells), K8 (luminal cells), NGFR (basal cells) and acetylated tubulin (ciliated cells). | Pseudostratified basal cell layer and luminal cells --> thinned basal cells and ciliated cells |  | [18] |
|  |  |  |  |  |  |  |  |  |  |
| Esophagus | Clonal from pediatric esophageal biopsies and murine esophagus | #5 | aDMEM/F12 | Glutamine, HEPES, Bovine pituitary extract, N2, B27, N-Acetyl Cysteine, Gastrin, Nicotinamide, Calcium Chloride | EGF, Noggin/R-Spondin1, Wnt3A, A83-01, SB202190, Y27632 | ICC for tracheal epithelial differentiation (IVL); PCR for IVL, FLG, NOTCH pathway | Stratified squamous spheroid with abundant shedding | Response to inflammatory molecules and cytokines. Used to model gastroesophageal reflux disease and eosinophilic esophagitis | [19] |
| Esophagus | hESCs-> Anterior foregut spheroids -> 3D culture | #1 | DMEM/F12 | B27, N2, HEPES, Glutamine | EGF, (Noggin, FGF10, CultureOne supplement for first three days) | ICC for tracheal markers (CDH1, SOX2, PAX9, NKX2.1), keratinocytes (KRT4/14/13).basal marker (p63). PCR for MNX1, IVL, CRNN, p63, KRT5/13. Transcriptome benchmarked to adult esophagus and other adult tissues. | Stratified squamous spheroid | Investigated the role of SOX2, BMP and WNTs in esophageal atresia | [20] |
| Esophagus | hESCs, hiPSCs -> ITGB4+ EPCAM+ Esophageal progenitor cells | #4 | IMDM+F12 | Bovine Albumin FractionV, Glutamax, N2, B27, Ascorbic acid, MTG | Y27632, Noggin, SB431542, CHIR99021, FGF2, EGF | qPCR and ICC for tracheal markers (PAX9,FOXE1, FOXA2, SOX2, PROX1, HNF6, NKX2.1, SOX9), Keratinocytes (KRT5/7/13), epithelia (EPCAM), basal(p63, ITGB4), and differentiating cells (IVL, Loricrin) all benchmarked to fetal human and mouse lung. | Stratified squamous spheroid | - | [21] |
|  |  |  |  |  |  |  |  |  |  |
| Pancreas | Mouse fetal progenitors | #5 Matrigel, different media for spheres and organoids | Organoid: DMEM/F12, Pen/Strep, beta-mercaptoethanol. Spheres: DMEM/F12, Pen/Strep | Organoid: KOSR. Spheroid: B27. | Organoid: phorbol myristate acetate, ROCKi, R-spondin1, FGF10, FGF1, heparin. Spheroid: ROCKi, FGF2. | ICC for epithelial (Ecad), Progenitor (Pdx1, Sox9, Hnf1b), ductal/progenitor (Muc1, DBA lectin, aPKC), acinar (Amylase, Ptf1a), endocrine progenitor (Neurog3) and endocrine cells (Ins, Gcg, Pcsk1/3, c-peptide 1/2). PCR for several markers above and single cell PCR for 42 markers benchmarked to E10.5 pancreas. | Two culture media lead to spheres or organoids. The latter exhibit extensive morphogenesis. Network of ducts which does not evolve into a tree like the neonate pancreas, peripheral acinar cells and interspersed endocrine cells. | Integrates to fetal pancreas upon transplantation | [22,23] |
| Pancreas | Pancreatic-ductal-ligation injury induced Lgr5+ ductal cells in mouse | #5 Matrigel | AdDMEM/F12 | N-Acetylcysteine, gastrin, nicotinamide | EGF, RSPO-1, Noggin, FGF10 | ICC for CK, MIC1-1C3, Sox9 (ductal) and PDX1 (progenitors). Aggregation of adult dissociated pancreas with embryonic dissociated pancreas and transplant to mouse supports ductal cell engraftment. Some may have a progenitor identity and differentiate to a beta like cell after in vivo engraftment | Single-layered epithelium with a duct-like phenotype. Mostly spheres, some seemed to have small budding at the periphery of the sphere. |  | [24] |
| Pancreas | Whole adult mouse pancreas dissociated to single cells (likely only ductal cells formed organoids), and CD133+/Sox9+ (ductal) cells sorted from dissociated adult mouse pancreas | #5 Matrigel (-/+ RSPO1) or laminin hydrogel | DMEM/F12, methylcellulose, murine embryonic stem cell pancreatic like cell conditioned medium | FCS, nicotinamide | actinvin-betaB, exendin-4, VEGF-A, RSPO-1, Dkk1 (Wnt inhibitor) | Spheres grown in Matrigel (-RSPO1): ductal cells, progenitors, endocrine, acinar. Spheres grown in Matrigel (+RSPO1): ductal cells, progenitors (increased expression of progenitor markers in dense colonies). Spheres grown in laminin: endocrine cells, acinar cells. qRT-PCR for Sox9, Mucin1 (ductal cells), Pdx1, Nkx6.1 (progenitors), Ngn3 (endocrine progenitors), insulin1, insulin2, glucagon (endocrine cells), elastase1 (acinar cells) in ring, dense, and "endo/acinar" colonies. IHC for Sox9, Mucin1 (ductal cells), Pdx1 (progenitors), amylase (acinar cells), C-peptide (endocrine cells) in ring colonies. IHC for Sox9 (ductal) and Ngn3 (endocrine progenitor) in dense colonies. IHC for C-peptide, glucagon (endocrine cells) and amylase (acinar cells) in endo/acinar colonies. | spheroid: "ring" (-RSPO1) and "dense" (+RSPO1). | Endocrine/acinar colonies secrete C-peptide in response to D-glucose (no benchmarking to freshly isolated islets) | [25] |
| Pancreas | hESCs, hiPSCs -> PDX1+ NKX6-1+ progenitor cells | #2 | Embedded medium: BCDB131, sodium-bicarbonate Suspension Medium: DMEM/F12, beta-mercaptoethanol | Embedded medium: glucose, FAF-BSA, glutamine, ascorbic acid, ITS-X. EM day 18: nicotinamide. Suspension medium: KOSR | EM day 12: +ROCKi. EM day 14: ROCKi, FGF2. EM day 18: FGF2. Suspension medium: FGF10, EGF2, CHIR99021, phorbol myristate acetate. | ICC for Ecad, PDX1, SOX9, NKX6.1 (progenitors), amylase, chymotrypsin C (acinar), SOX9, cytokeratin 19 (ductal);  Electron microscopy of acinar and ductal structures. | Sphere with central lumen, basal lamina and apical-basal polarity,   ducts have microvilli and secretary granules in acinar cells. Some conditions more organoid-like. | Enzymatic assay for carbonic anhydrase activity in ductal cells similar to freshly isolated cells, and activity of amylase, trypsin and elastase detected. Modeling cystic fibrosis. | [26] |
| Pancreas | hESCs -> PDX1+ NKX6-1+ cells | #2 | DMEM | PTOM: B27, ascorbic acid, hydrocortisone. POMM: B27, ascorbic acid. PODM 1 & 2: B27, 2-phosphoascorbic acid. | PTOM: insulin, FGF2, all trans retinoic acid, ROCKi. PODM 1: FGF7, EGF, ALK5i, y-secretase inhibitor. PODM 2" FGF7, EGF. | Microarray for Pdx1, Nkx6.1, Sox9 (progenitors), Gata4 (acinar cells). IHC for PDX1, NKX6.1, SOX9 (progenitors), PTF1A (progenitors or acinar cells), KRT19 (ductal cells). qPCR for Cpa1 (progenitor or acinar cells), CA2 (ductal cells) | Sphere | Modeling cancer | [27] |
| Pancreas | hiPSCs and mouse E14.5 pancreatic cells | #2 | MCDB131 | Glutamax, BSA, ITS-X, | SANT-1, Retinoic acid, LDN193189, TPB | ICC for polarity markers (aPKC, Ezrin, EBP50, and PARD6B) in progenitor spheroids. SC-RNAseq Immunostaining for endocrine markers: NGN3, NEUROD1, CGA, Ins and GCG | Sphere: polarised pancreatic epithelia with lumen. delaminating endocrine committed cells. |  | [28] |
| Pancreatic Islet | Procr+ cells sorted from adult mouse islets | #1 &3 2D Matrigel coating + co-culture with endothelial cells | DMEM/F12, Pen/Strep | B27 | ITS, EGF, heparin, FGF2, VEGFa | qPCR for Ins, Mafa, Ucn3, Pdx1 (beta cells). IHC for Ins (beta cells), Gcg (alpha cells), Sst (delta cells), Ppy (pancreatic polypeptide cells). Compared to freshly isolated islets. | Dense spheres with infiltration by endothelial cells | Islet organoids secrete insulin and glucagon in vitro in response to glucose. Transplant of islet organoids into diabetic mice reduces blood sugar and secrete insulin in response to glucose. | [29] |
|  |  |  |  |  |  |  |  |  |  |
| Liver | hiPSCs -> hepatoblasts | #3 iPSC- derived hepatic cells + HUVECs + hMSCs | DMEM/F12 | KoSR, Glutamine, non-essential amino acids, 2-mercaptoethanol, DMSO | Oncostatin M, HGF | ICC for AFP (hepatoblasts), microarray analysis comparing with mouse fetal and adult livers. Also contains mesenchymal and endothelial progenitors. | Organoid | Develops into functional system upon transplant and produces albumin | [30,31] |
| Liver | Mouse CCl4 injured bile ducts or Lgr5+ cells sorted from CCl4 injured bile ducts. Extension to EpCam+ human liver cells. | #5 Matrigel | AdDMEM/F12 | B27, N2, N-acetylcysteine, gastrin, nicotinamide | EGF, RSPO1 conditioned medium, Fgf10, HGF. For human Forskolin, A83-01, Noggin. | Expansion medium favors progenitors and bile duct cells, vs. differentiation medium promotes hepatocytes at the expense of progenitors (bile duct cells still present). Microarray for Lgr5, Sox9, CD44, Prom1 (progenitors). qPCR for Ttr, Glul, Hnf1a, Hnf4a (hepatocytes) and Hnf6, Krt19, Krt7 (bile duct markers). ICC for Hnf4-alpha, albumin, OC2-2F8 (hepatocytes) and Krt19 (bile duct cells). | Spheres and some more complex structures | Hepatocytes can take up low density lipoproteins, store glycogen, secrete albumin into the lumen, and induce cytochrome p450 function but to a lesser extent than freshly isolated hepatocytes. Transplant of organoids grown in differentiation media into Fah-/- mice allowed full maturation of hepatocytes and contribute to liver function in vivo. | [32,33] |
| Liver | Dissociated whole liver from adult mice | #5 Matrigel | DMEM/F12 | N2, B27 without vitamin A, nicotinamide, dexamethasone, HEPES | Y27632, EGF, HGF, Wnt3a, Rspo1 | IHC for Sox9, CK19, HNF1b (bile duct cells) and HNF4a (hepatocytes). Microarray for Afp, Abcc2, Prox1 (progenitor/hepatoblast); Krt19, G6pc3, Krt7, Sox9, Hnf1b (bile duct cells), Cldn3, Onecut1, Onecut2 (hepatocyte) | Spheres | Translpant into FAH-/- mice | [34] |
| Liver | Hepatocytes from adult mouse liver | #5 Matrigel | William's E medium | Glutamax, Non-essential amino acids, normocin, B27, N2, nicotinamide, N-acetylcysteine, Y-27632, A83-01, CHIR99021 | EGF, HGF, TNFalpha  For long term culture: Noggin | ICC for HNF4a (hepatocytes) and CD26+ bile duct cells | Organoids with polygonal cells forming pseudo-glandular rosettes with lumen-like structures". Formation of bile canaliculi based on CD26 staining | Hepatocyte function observed by albumin secretion, CYP3A11 enzymatic activity, uptake of low-density-lipoproteins. Bile canaliculi function observed by uptake of fluorescent dye and accumulation in lumens. Transplant of intact 3D hepatocyte colonies into FAH-/- mice. | [35] |
| Liver | Hepatocytes from adult mouse and human liver, includes sorting and clonogenicity  Human fetal hepatocytes | #5 Matrigel | AdDMEM/F12 | B27 (-VitA), N-acetylcysteine, Nicotinamide | RSPO1, EGF, gastrin, CHIR99021, HGF, FGF7, FGF10 , A83-01, Y-27632  (+ TGFa for human fetal hepatocytes). For differentiation Dexamethasone & Oncostatin) | ICC for Albumin, Cyp2E1, HNF4a, absence of KRT19 & 7. qPCR for multiple hepatocyte markers and transcriptome compared to cholangiocyte spheres and hepatocytes in vivo. Single cell sequencing (not benchmarked to tissue in vivo). EM. | Networks of bile canaliculi | LDL uptake and glycogen storage, transport of Rhodamine123 into lumen. Albumin, AFP & A1AT secretion. CYP3A4 activity. Repopulation of FAH-/- mice. | [36] |
|  |  |  |  |  |  |  |  |  |  |
| Bile duct | hESCs, hiPSCs -> hepatoblasts -> coculture with OP9 cells | #3 | H21 MEDM/Ham's F12 | BSA, B27, ascorbic acid, gluatmine, monothiolglycerol | HGF, EGF, TGF-beta, y-secretase inhibitor | Cholangiocytes (ductal cells) shown by ICC for KRT9 and CFTR | Tubular, cystic, mixed tubular/cystic, or dense spherical morphology, mature polarised cells observed in tubular and cystic colonies | Efflux of rhodamine dye into the lumen and activity of CFTR by forskolin swelling assay | [37] |
| Bile duct | hiPSCs ->hepatoblasts ->cholangiocyte progenitors | #2 | William's E medium, Pen/Strep | nicotinamide, sodium bicarbonate, ascorbic acid, sodium pyruvate, glucose, HEPES, dexamehtasone, Glutamax, | ITS+premix, EGF | Cholangiocytes shown by qPCR for CK7, CK19, HNF1B, GGT1, JAG1, NOTCH2, CFTR, SCR, SSTR2, AQP1, AE2, compared with primary cholangiocytes. ICC for HNF1B, CK7, CK18, CK19 (biliary cells) (only showed IHC data for spheres, qPCR unknown) | Cystic organoid and branched/ tubular organoids | Secretion of Rhodamine dye into the lumen, export of fluorescent bile-acid salt from the lumen. Activated calcium signaling in response to acetylcholine and ATP, proliferation in response to VEGF stimulation, and GGP and ALP activities similar to primary cholangiocytes | [38] |
|  |  |  |  |  |  |  |  |  |  |
| Prostate | Murine prostate epithelial cells | #5 | Prostate epithelial growth medium (serum free) | BPE, Triiodothyronine, hydrocortisone, transferrin, Retinoic acid, GA-1000 | Insulin, hEGF, epinephrine | ICC shows outer basal cells (TP63, CK5), luminal layer (CK8) and qPCR for CD24, CD49f, and Sca-1, CK5, TP63, low CK8, no synaptophysin. AR only in the presence of androgens. No Nkx3.1. | Spheres with luminal and basal layers. Subcutaneous grafts with urogenital mesenchyme. | Response to androgens | [39] |
| Prostate | Human prostactomy: (Trop2), CD44, and CD49f basal preferentially form spheres clonally | #5 | Prostate epithelial growth medium (serum free) | BPE, Triiodothyronine, hydrocortisone, transferrin, Retinoic acid, GA-1000 | Insulin, hEGF, epinephrine | ICC for basal cells (CD44, CD49f, CK5, TP63), possibly Luminal cells, low CK8 but no PSA. AR only in the presence of androgens. No Nkx3.1. No neuroendocrine cells (no synaptophysin, no ChgA) | Spheres with basal layer and a luminal layer after subcutaneous engraftment with urogenital mesenchyme. | Tumor modelling: Though derived from tumor tissues, cells with a TMPRSS-ERG fusion were counterselected | [40] |
| Prostate | Murine (anterior, dorsolateral and ventral) and human prostate, single basal cells (CD49f) and single luminal cells (CD26+) with low efficiency | #5 Matrigel | ADMEM/F12 | mouse: B27, HEPES, Glutamax  human: in addition to mouse medium, SB202190, nicotinamide | mouse: EGF, R-spondin1, Noggin, TGF-beta/A83-01, dihydrotestosterone  For human: in addition to mouse medium, FGF10, FGF2, ProstaglandinE2 | ICC shows outer basal (CK5, TP63), inner luminal layers (CK8, NKX3.1, PSA, AR). qPCR for AR targets (Fkbp5, Psca). | Spheres with luminal and basal layers. Kidney capsule grafts with urogenital mesenchyme. | Response to androgens; PTEN inactivation as a model of prostate cancer | [41] |
| Prostate | Mouse adult prostate luminal cells (NKX3.1+ or CK8 or cK18) better than basal (CK5) and human prostatectomy samples | #5 Matrigel | Hepatocyte culture medium (corning) | Glutamax, 5% Matrigel (for suspension culture only), 5% charcoal-stripped FBS | EGF, Y-27632, DHT | ICC shows outer basal (CK5, TP63), inner luminal layers (CK8, NKX3.1, AR) and FOXA1. qPCR for *Fkbp5, Mm, Psca, Igfbp3*. | Spheres with luminal and basal layers. Kidney capsule transplantation | Response to androgens; Prostate cells from NKX3.1-/-;NKX3.1+/- with PTEN+/-; TRAMP Tg; Ptenfl/fl; p53fl/fl prostate tumor models generate more organoids and filled spheroid structures. Tumor induction in culture possible. | [42] |
| Prostate | EPCAM+ murine and human prostate | #5 Matrigel | ADMEM/F12 | Glutamine, glucose | mouse: EGF, bFGF, LONG R3 IGF-1, holo-transferrin, insulin  for human: in addition to mouse medium, progesterone and sodium selenite | ICC showing outer basal (CK5, TP63), inner luminal layers (CK8, NKX3.1). FACS for SCA-1, CD49f, TROP2. Transcriptome comparing expanding and differentiating spheres and benchmarking to tissue in vivo for a few markers by ICC | Spheres with luminal and basal layers. Kidney capsule transplantation | - | [43] |
| Prostate | hiPSCs + rat urogenital mesenchyme | #3 rat UGM cells | RPMI1640 | rat collagen matrix, FBS |  | ICC showing outer basal (TP63), inner luminal layers (CK8/CK18, NKX3.1), AR, rare endocrine cells (CHGA). Some mesenchyme. Transcriptome and comparison to human prostate (inflammation signature). | Spheres. Tubular structures with dense bud tips on long term. Luminal and basal layers. | - | [44] |
|  |  |  |  |  |  |  |  |  |  |
| Bladder | Murine Shh+  urothelial cells | #5 Suspension Matrigel | DMEM | Conditioned medium: V79 lung fibroblast-derived cells in DMEM with 5% FBS | EGF | ICC shows outer basal cells expressing epithelial markers Ck5, Shh and inner luminal layers that lack Ck5 expression | Spheres with luminal and basal layers |  | [45] |
|  |  |  |  |  |  |  |  |  |  |
| Salivary | E13 mouse submandibular gland (whole epithelia or rudiments) | #5 & 3 Matrigel, with or without mesenchyme | DMEM/F12 |  | EGF, FGF7 | Time lapse imaging with GFP labeled end bud cells. ICC for epithelial markers (E-cad) and others (Actin, beta-catenin), qPCR for proacinar and terminal tubule cell markers (AQP5, PIP, SMGC). Fetal. | Branching morphogenesis to form glandular structure, no ductal or acinar cells but have lumen. |  | [46] [47] |
| Salivary | Mouse and human submandibular cells in clumps | In suspension | DMEM/F-12 | Glutamax, N2, Insulin, dexamethasone | EGF, FGF2 | ICC for acinar markers (PAS, amylase), submandibular gland ductal cell marker (CK7, CK14), ductal stem cell marker (Sca-1, c-Kit, Msi-1). RT-PCR for amylase | Ductal structure when embedded in 3D collagen | Restored organ function by transplanted progenitor/stem cells after irradiation in mice | [48,49] |
| Salivary | dissociated human nonmalignant submandibular cells | #5 Matrigel | DMEM/F12 | Fetal calf serum, Glutamax, N2, dexamethasone | EGF, FGF2, insulin | ICC for AQP-5, alpha-amylase (acinar cells) and Cytokeratin (ductal cells) | Branched, lobular structure | Transplant of human organoids into mice with irradiated salivary glands restored saliva production, with long term engraftment. | [50] |
|  |  |  |  |  |  |  |  |  |  |
| Thyroid | Ectopic Nkx2-1/Pax8 expressing mESCs | #4 with ectopic expression of Nkx2-1 & Pax8 | DMEM | FBS, essential amino acids, sodium pyruvate b-mercaptoethanol, ascorbic acid | TSH | ICC and qPCR for thyroid follicle markers (TG, TG-I), basolateral (NIS), apical (ZO1) | Follicular aggregates | iodide organification upon TSH treatment. transplantation of follicles into mouse with hypothyroidism -> functioanl rescue | [51] |
| Thyroid | mESCs -> Nkx2-1+ progenitors | #4  DE as clumps  on Matrigel | differentiation medium: IMDM + Ham's modified F12 medium  maturation media: Ham's F12 | differentiation medium: N2, B27, RA, BSA, Glutamine, ascorbic acid, monothioglycerol, heparin   maturation medium: HEPES, CaCl2, heparin, BSA, ITS, dexamethasone | differentiation medium: FGF2, FGF10, IGF-1, EGF, insulin, bTSH   maturation medium: IGF-1, insulin, EGF, bTSH | qPCR for thyroid genes Nkx2.1, Pax8, thyroglobulin, Tsh-r, Tpo, Nis. Immunostaining for NKX2.1, Pax8; in-vivo loss of function of BMP receptor and FGF receptor in *Xenopus* | Thyroid follicles: monolayered Nkx2.1+ Pax8+ epithelia around lumen filled with Thyroglobulin. | Incorporation of iodine into thyroglobulin to produce T4 hormone. Transplant into hypothyroid mice restores circulating T4 hormone and lowers TSH plasma levels | [52] |

**References**

1. Sato T, Vries RG, Snippert HJ, van de Wetering M, Barker N, Stange DE, van Es JH, Abo A, Kujala P, Peters PJ, Clevers H (2009) Single Lgr5 stem cells build crypt-villus structures in vitro without a mesenchymal niche. Nature 459 (7244):262-265. doi:10.1038/nature07935

2. Sato T, van Es JH, Snippert HJ, Stange DE, Vries RG, van den Born M, Barker N, Shroyer NF, van de Wetering M, Clevers H (2011) Paneth cells constitute the niche for Lgr5 stem cells in intestinal crypts. Nature 469 (7330):415-418. doi:10.1038/nature09637

3. Serra D, Mayr U, Boni A, Lukonin I, Rempfler M, Challet Meylan L, Stadler MB, Strnad P, Papasaikas P, Vischi D, Waldt A, Roma G, Liberali P (2019) Self-organization and symmetry breaking in intestinal organoid development. Nature 569 (7754):66-72. doi:10.1038/s41586-019-1146-y

4. Fordham RP, Yui S, Hannan NR, Soendergaard C, Madgwick A, Schweiger PJ, Nielsen OH, Vallier L, Pedersen RA, Nakamura T, Watanabe M, Jensen KB (2013) Transplantation of expanded fetal intestinal progenitors contributes to colon regeneration after injury. Cell Stem Cell 13 (6):734-744. doi:10.1016/j.stem.2013.09.015

5. Spence JR, Mayhew CN, Rankin SA, Kuhar MF, Vallance JE, Tolle K, Hoskins EE, Kalinichenko VV, Wells SI, Zorn AM, Shroyer NF, Wells JM (2011) Directed differentiation of human pluripotent stem cells into intestinal tissue in vitro. Nature 470 (7332):105-109. doi:10.1038/nature09691

6. Mithal A, Capilla A, Heinze D, Berical A, Villacorta-Martin C, Vedaie M, Jacob A, Abo K, Szymaniak A, Peasley M, Stuffer A, Mahoney J, Kotton DN, Hawkins F, Mostoslavsky G (2020) Generation of mesenchyme free intestinal organoids from human induced pluripotent stem cells. Nat Commun 11 (1):215. doi:10.1038/s41467-019-13916-6

7. Jung P, Sato T, Merlos-Suarez A, Barriga FM, Iglesias M, Rossell D, Auer H, Gallardo M, Blasco MA, Sancho E, Clevers H, Batlle E (2011) Isolation and in vitro expansion of human colonic stem cells. Nat Med 17 (10):1225-1227. doi:10.1038/nm.2470

8. Munera JO, Sundaram N, Rankin SA, Hill D, Watson C, Mahe M, Vallance JE, Shroyer NF, Sinagoga KL, Zarzoso-Lacoste A, Hudson JR, Howell JC, Chatuvedi P, Spence JR, Shannon JM, Zorn AM, Helmrath MA, Wells JM (2017) Differentiation of Human Pluripotent Stem Cells into Colonic Organoids via Transient Activation of BMP Signaling. Cell Stem Cell 21 (1):51-64 e56. doi:10.1016/j.stem.2017.05.020

9. Barker N, Huch M, Kujala P, van de Wetering M, Snippert HJ, van Es JH, Sato T, Stange DE, Begthel H, van den Born M, Danenberg E, van den Brink S, Korving J, Abo A, Peters PJ, Wright N, Poulsom R, Clevers H (2010) Lgr5(+ve) stem cells drive self-renewal in the stomach and build long-lived gastric units in vitro. Cell Stem Cell 6 (1):25-36. doi:10.1016/j.stem.2009.11.013

10. Stange DE, Koo BK, Huch M, Sibbel G, Basak O, Lyubimova A, Kujala P, Bartfeld S, Koster J, Geahlen JH, Peters PJ, van Es JH, van de Wetering M, Mills JC, Clevers H (2013) Differentiated Troy+ chief cells act as reserve stem cells to generate all lineages of the stomach epithelium. Cell 155 (2):357-368. doi:10.1016/j.cell.2013.09.008

11. McCracken KW, Cata EM, Crawford CM, Sinagoga KL, Schumacher M, Rockich BE, Tsai YH, Mayhew CN, Spence JR, Zavros Y, Wells JM (2014) Modelling human development and disease in pluripotent stem-cell-derived gastric organoids. Nature 516 (7531):400-404. doi:10.1038/nature13863

12. Hawkins F, Kramer P, Jacob A, Driver I, Thomas DC, McCauley KB, Skvir N, Crane AM, Kurmann AA, Hollenberg AN, Nguyen S, Wong BG, Khalil AS, Huang SX, Guttentag S, Rock JR, Shannon JM, Davis BR, Kotton DN (2017) Prospective isolation of NKX2-1-expressing human lung progenitors derived from pluripotent stem cells. J Clin Invest 127 (6):2277-2294. doi:10.1172/JCI89950

13. Jacob A, Morley M, Hawkins F, McCauley KB, Jean JC, Heins H, Na CL, Weaver TE, Vedaie M, Hurley K, Hinds A, Russo SJ, Kook S, Zacharias W, Ochs M, Traber K, Quinton LJ, Crane A, Davis BR, White FV, Wambach J, Whitsett JA, Cole FS, Morrisey EE, Guttentag SH, Beers MF, Kotton DN (2017) Differentiation of Human Pluripotent Stem Cells into Functional Lung Alveolar Epithelial Cells. Cell Stem Cell 21 (4):472-488 e410. doi:10.1016/j.stem.2017.08.014

14. Dye BR, Hill DR, Ferguson MA, Tsai YH, Nagy MS, Dyal R, Wells JM, Mayhew CN, Nattiv R, Klein OD, White ES, Deutsch GH, Spence JR (2015) In vitro generation of human pluripotent stem cell derived lung organoids. Elife 4. doi:10.7554/eLife.05098

15. Lee JH, Bhang DH, Beede A, Huang TL, Stripp BR, Bloch KD, Wagers AJ, Tseng YH, Ryeom S, Kim CF (2014) Lung stem cell differentiation in mice directed by endothelial cells via a BMP4-NFATc1-thrombospondin-1 axis. Cell 156 (3):440-455. doi:10.1016/j.cell.2013.12.039

16. Nikolic MZ, Caritg O, Jeng Q, Johnson JA, Sun D, Howell KJ, Brady JL, Laresgoiti U, Allen G, Butler R, Zilbauer M, Giangreco A, Rawlins EL (2017) Human embryonic lung epithelial tips are multipotent progenitors that can be expanded in vitro as long-term self-renewing organoids. Elife 6. doi:10.7554/eLife.26575

17. Miller AJ, Hill DR, Nagy MS, Aoki Y, Dye BR, Chin AM, Huang S, Zhu F, White ES, Lama V, Spence JR (2018) In Vitro Induction and In Vivo Engraftment of Lung Bud Tip Progenitor Cells Derived from Human Pluripotent Stem Cells. Stem Cell Reports 10 (1):101-119. doi:10.1016/j.stemcr.2017.11.012

18. Rock JR, Onaitis MW, Rawlins EL, Lu Y, Clark CP, Xue Y, Randell SH, Hogan BL (2009) Basal cells as stem cells of the mouse trachea and human airway epithelium. Proc Natl Acad Sci U S A 106 (31):12771-12775. doi:10.1073/pnas.0906850106

19. Kasagi Y, Chandramouleeswaran PM, Whelan KA, Tanaka K, Giroux V, Sharma M, Wang J, Benitez AJ, DeMarshall M, Tobias JW, Hamilton KE, Falk GW, Spergel JM, Klein-Szanto AJ, Rustgi AK, Muir AB, Nakagawa H (2018) The Esophageal Organoid System Reveals Functional Interplay Between Notch and Cytokines in Reactive Epithelial Changes. Cell Mol Gastroenterol Hepatol 5 (3):333-352. doi:10.1016/j.jcmgh.2017.12.013

20. Trisno SL, Philo KED, McCracken KW, Cata EM, Ruiz-Torres S, Rankin SA, Han L, Nasr T, Chaturvedi P, Rothenberg ME, Mandegar MA, Wells SI, Zorn AM, Wells JM (2018) Esophageal Organoids from Human Pluripotent Stem Cells Delineate Sox2 Functions during Esophageal Specification. Cell Stem Cell 23 (4):501-515 e507. doi:10.1016/j.stem.2018.08.008

21. Zhang Y, Yang Y, Jiang M, Huang SX, Zhang W, Al Alam D, Danopoulos S, Mori M, Chen YW, Balasubramanian R, Chuva de Sousa Lopes SM, Serra C, Bialecka M, Kim E, Lin S, Toste de Carvalho ALR, Riccio PN, Cardoso WV, Zhang X, Snoeck HW, Que J (2018) 3D Modeling of Esophageal Development using Human PSC-Derived Basal Progenitors Reveals a Critical Role for Notch Signaling. Cell Stem Cell 23 (4):516-529 e515. doi:10.1016/j.stem.2018.08.009

22. Greggio C, De Franceschi F, Figueiredo-Larsen M, Gobaa S, Ranga A, Semb H, Lutolf M, Grapin-Botton A (2013) Artificial three-dimensional niches deconstruct pancreas development in vitro. Development 140 (21):4452-4462. doi:10.1242/dev.096628

23. Dahl-Jensen SB, Yennek S, Flasse L, Larsen HL, Sever D, Karremore G, Novak I, Sneppen K, Grapin-Botton A (2018) Deconstructing the principles of ductal network formation in the pancreas. PLoS Biol 16 (7):e2002842. doi:10.1371/journal.pbio.2002842

24. Huch M, Bonfanti P, Boj SF, Sato T, Loomans CJ, van de Wetering M, Sojoodi M, Li VS, Schuijers J, Gracanin A, Ringnalda F, Begthel H, Hamer K, Mulder J, van Es JH, de Koning E, Vries RG, Heimberg H, Clevers H (2013) Unlimited in vitro expansion of adult bi-potent pancreas progenitors through the Lgr5/R-spondin axis. EMBO J 32 (20):2708-2721. doi:10.1038/emboj.2013.204

25. Jin L, Feng T, Shih HP, Zerda R, Luo A, Hsu J, Mahdavi A, Sander M, Tirrell DA, Riggs AD, Ku HT (2013) Colony-forming cells in the adult mouse pancreas are expandable in Matrigel and form endocrine/acinar colonies in laminin hydrogel. Proc Natl Acad Sci U S A 110 (10):3907-3912. doi:10.1073/pnas.1301889110

26. Hohwieler M, Illing A, Hermann PC, Mayer T, Stockmann M, Perkhofer L, Eiseler T, Antony JS, Muller M, Renz S, Kuo CC, Lin Q, Sendler M, Breunig M, Kleiderman SM, Lechel A, Zenker M, Leichsenring M, Rosendahl J, Zenke M, Sainz B, Jr., Mayerle J, Costa IG, Seufferlein T, Kormann M, Wagner M, Liebau S, Kleger A (2017) Human pluripotent stem cell-derived acinar/ductal organoids generate human pancreas upon orthotopic transplantation and allow disease modelling. Gut 66 (3):473-486. doi:10.1136/gutjnl-2016-312423

27. Huang L, Holtzinger A, Jagan I, BeGora M, Lohse I, Ngai N, Nostro C, Wang R, Muthuswamy LB, Crawford HC, Arrowsmith C, Kalloger SE, Renouf DJ, Connor AA, Cleary S, Schaeffer DF, Roehrl M, Tsao MS, Gallinger S, Keller G, Muthuswamy SK (2015) Ductal pancreatic cancer modeling and drug screening using human pluripotent stem cell- and patient-derived tumor organoids. Nat Med 21 (11):1364-1371. doi:10.1038/nm.3973

28. Bakhti M, Scheibner K, Tritschler S, Bastidas-Ponce A, Tarquis-Medina M, Theis FJ, Lickert H (2019) Establishment of a high-resolution 3D modeling system for studying pancreatic epithelial cell biology in vitro. Mol Metab 30:16-29. doi:10.1016/j.molmet.2019.09.005

29. Wang D, Wang J, Bai L, Pan H, Feng H, Clevers H, Zeng YA (2020) Long-Term Expansion of Pancreatic Islet Organoids from Resident Procr(+) Progenitors. Cell 180 (6):1198-1211 e1119. doi:10.1016/j.cell.2020.02.048

30. Takebe T, Sekine K, Enomura M, Koike H, Kimura M, Ogaeri T, Zhang RR, Ueno Y, Zheng YW, Koike N, Aoyama S, Adachi Y, Taniguchi H (2013) Vascularized and functional human liver from an iPSC-derived organ bud transplant. Nature 499 (7459):481-484. doi:10.1038/nature12271

31. Camp JG, Sekine K, Gerber T, Loeffler-Wirth H, Binder H, Gac M, Kanton S, Kageyama J, Damm G, Seehofer D, Belicova L, Bickle M, Barsacchi R, Okuda R, Yoshizawa E, Kimura M, Ayabe H, Taniguchi H, Takebe T, Treutlein B (2017) Multilineage communication regulates human liver bud development from pluripotency. Nature 546 (7659):533-538. doi:10.1038/nature22796

32. Huch M, Dorrell C, Boj SF, van Es JH, Li VS, van de Wetering M, Sato T, Hamer K, Sasaki N, Finegold MJ, Haft A, Vries RG, Grompe M, Clevers H (2013) In vitro expansion of single Lgr5+ liver stem cells induced by Wnt-driven regeneration. Nature 494 (7436):247-250. doi:10.1038/nature11826

33. Huch M, Gehart H, van Boxtel R, Hamer K, Blokzijl F, Verstegen MM, Ellis E, van Wenum M, Fuchs SA, de Ligt J, van de Wetering M, Sasaki N, Boers SJ, Kemperman H, de Jonge J, Ijzermans JN, Nieuwenhuis EE, Hoekstra R, Strom S, Vries RR, van der Laan LJ, Cuppen E, Clevers H (2015) Long-term culture of genome-stable bipotent stem cells from adult human liver. Cell 160 (1-2):299-312. doi:10.1016/j.cell.2014.11.050

34. Yimlamai D, Christodoulou C, Galli GG, Yanger K, Pepe-Mooney B, Gurung B, Shrestha K, Cahan P, Stanger BZ, Camargo FD (2014) Hippo pathway activity influences liver cell fate. Cell 157 (6):1324-1338. doi:10.1016/j.cell.2014.03.060

35. Peng WC, Logan CY, Fish M, Anbarchian T, Aguisanda F, Alvarez-Varela A, Wu P, Jin Y, Zhu J, Li B, Grompe M, Wang B, Nusse R (2018) Inflammatory Cytokine TNFalpha Promotes the Long-Term Expansion of Primary Hepatocytes in 3D Culture. Cell 175 (6):1607-1619 e1615. doi:10.1016/j.cell.2018.11.012

36. Hu H, Gehart H, Artegiani B, C LO-I, Dekkers F, Basak O, van Es J, Chuva de Sousa Lopes SM, Begthel H, Korving J, van den Born M, Zou C, Quirk C, Chiriboga L, Rice CM, Ma S, Rios A, Peters PJ, de Jong YP, Clevers H (2018) Long-Term Expansion of Functional Mouse and Human Hepatocytes as 3D Organoids. Cell 175 (6):1591-1606 e1519. doi:10.1016/j.cell.2018.11.013

37. Ogawa M, Ogawa S, Bear CE, Ahmadi S, Chin S, Li B, Grompe M, Keller G, Kamath BM, Ghanekar A (2015) Directed differentiation of cholangiocytes from human pluripotent stem cells. Nat Biotechnol 33 (8):853-861. doi:10.1038/nbt.3294

38. Sampaziotis F, de Brito MC, Madrigal P, Bertero A, Saeb-Parsy K, Soares FAC, Schrumpf E, Melum E, Karlsen TH, Bradley JA, Gelson WT, Davies S, Baker A, Kaser A, Alexander GJ, Hannan NRF, Vallier L (2015) Cholangiocytes derived from human induced pluripotent stem cells for disease modeling and drug validation. Nat Biotechnol 33 (8):845-852. doi:10.1038/nbt.3275

39. Xin L, Lukacs RU, Lawson DA, Cheng D, Witte ON (2007) Self-renewal and multilineage differentiation in vitro from murine prostate stem cells. Stem Cells 25 (11):2760-2769. doi:10.1634/stemcells.2007-0355

40. Garraway IP, Sun W, Tran CP, Perner S, Zhang B, Goldstein AS, Hahm SA, Haider M, Head CS, Reiter RE, Rubin MA, Witte ON (2010) Human prostate sphere-forming cells represent a subset of basal epithelial cells capable of glandular regeneration in vivo. Prostate 70 (5):491-501. doi:10.1002/pros.21083

41. Karthaus WR, Iaquinta PJ, Drost J, Gracanin A, van Boxtel R, Wongvipat J, Dowling CM, Gao D, Begthel H, Sachs N, Vries RGJ, Cuppen E, Chen Y, Sawyers CL, Clevers HC (2014) Identification of multipotent luminal progenitor cells in human prostate organoid cultures. Cell 159 (1):163-175. doi:10.1016/j.cell.2014.08.017

42. Chua CW, Shibata M, Lei M, Toivanen R, Barlow LJ, Bergren SK, Badani KK, McKiernan JM, Benson MC, Hibshoosh H, Shen MM (2014) Single luminal epithelial progenitors can generate prostate organoids in culture. Nat Cell Biol 16 (10):951-961, 951-954. doi:10.1038/ncb3047

43. Hofner T, Eisen C, Klein C, Rigo-Watermeier T, Goeppinger SM, Jauch A, Schoell B, Vogel V, Noll E, Weichert W, Baccelli I, Schillert A, Wagner S, Pahernik S, Sprick MR, Trumpp A (2015) Defined conditions for the isolation and expansion of basal prostate progenitor cells of mouse and human origin. Stem Cell Reports 4 (3):503-518. doi:10.1016/j.stemcr.2015.01.015

44. Hepburn AC, Curry EL, Moad M, Steele RE, Franco OE, Wilson L, Singh P, Buskin A, Crawford SE, Gaughan L, Mills IG, Hayward SW, Robson CN, Heer R (2020) Propagation of human prostate tissue from induced pluripotent stem cells. Stem Cells Transl Med. doi:10.1002/sctm.19-0286

45. Shin K, Lee J, Guo N, Kim J, Lim A, Qu L, Mysorekar IU, Beachy PA (2011) Hedgehog/Wnt feedback supports regenerative proliferation of epithelial stem cells in bladder. Nature 472 (7341):110-114. doi:10.1038/nature09851

46. Hosseini ZF, Nelson DA, Moskwa N, Larsen M (2019) Generating Embryonic Salivary Gland Organoids. Curr Protoc Cell Biol 83 (1):e76. doi:10.1002/cpcb.76

47. Wei C, Larsen M, Hoffman MP, Yamada KM (2007) Self-organization and branching morphogenesis of primary salivary epithelial cells. Tissue Eng 13 (4):721-735. doi:10.1089/ten.2006.0123

48. Feng J, van der Zwaag M, Stokman MA, van Os R, Coppes RP (2009) Isolation and characterization of human salivary gland cells for stem cell transplantation to reduce radiation-induced hyposalivation. Radiother Oncol 92 (3):466-471. doi:10.1016/j.radonc.2009.06.023

49. Lombaert IM, Brunsting JF, Wierenga PK, Faber H, Stokman MA, Kok T, Visser WH, Kampinga HH, de Haan G, Coppes RP (2008) Rescue of salivary gland function after stem cell transplantation in irradiated glands. PLoS One 3 (4):e2063. doi:10.1371/journal.pone.0002063

50. Pringle S, Maimets M, van der Zwaag M, Stokman MA, van Gosliga D, Zwart E, Witjes MJ, de Haan G, van Os R, Coppes RP (2016) Human Salivary Gland Stem Cells Functionally Restore Radiation Damaged Salivary Glands. Stem Cells 34 (3):640-652. doi:10.1002/stem.2278

51. Antonica F, Kasprzyk DF, Opitz R, Iacovino M, Liao XH, Dumitrescu AM, Refetoff S, Peremans K, Manto M, Kyba M, Costagliola S (2012) Generation of functional thyroid from embryonic stem cells. Nature 491 (7422):66-71. doi:10.1038/nature11525

52. Kurmann AA, Serra M, Hawkins F, Rankin SA, Mori M, Astapova I, Ullas S, Lin S, Bilodeau M, Rossant J, Jean JC, Ikonomou L, Deterding RR, Shannon JM, Zorn AM, Hollenberg AN, Kotton DN (2015) Regeneration of Thyroid Function by Transplantation of Differentiated Pluripotent Stem Cells. Cell Stem Cell 17 (5):527-542. doi:10.1016/j.stem.2015.09.004
